# High-flow nasal cannula in adults with acute respiratory failure and after extubation: a systematic review and meta-analysis

**DOI:** 10.1186/s12931-018-0908-7

**Published:** 2018-10-16

**Authors:** Zhiheng Xu, Yimin Li, Jianmeng Zhou, Xi Li, Yongbo Huang, Xiaoqing Liu, Karen E. A. Burns, Nanshan Zhong, Haibo Zhang

**Affiliations:** 1 State Key Laboratory of Respiratory Diseases, National Clinical Research Center for Respiratory Disease, Guangzhou Institute for Respiratory Health, Guangzhou, China; 2 Department of Critical Care Medicine, The First Affiliated Hospital of Guangzhou Medical University, Guangzhou, China; 3 Interdepartmental Division of Critical Care Medicine, University of Toronto, Toronto, ON Canada; 4 The Keenan Research Centre for Biomedical Science and the Li Ka Shing Knowledge Institute of St. Michael’s Hospital, Toronto, ON M5B1W8 Canada; 5 Departments of Anesthesia and Physiology, University of Toronto, Toronto, ON Canada

**Keywords:** Conventional oxygen therapy, Noninvasive ventilation, Extubation

## Abstract

**Background:**

High-flow nasal cannula (HFNC) can be used as an initial support strategy for patients with acute respiratory failure (ARF) and after extubation. However, no clear evidence exists to support or oppose HFNC use in clinical practice. We summarized the effects of HFNC, compared to conventional oxygen therapy (COT) and noninvasive ventilation (NIV), on important outcomes including treatment failure and intubation/reintubation rates in adult patients with ARF and after extubation.

**Methods:**

We searched 4 electronic databases (Pubmed, EMBASE, Scopus, and Web of Science) to identify randomized controlled trials (RCTs) comparing the effects of HFNC with either COT or NIV on rates of 1) treatment failure and 2) intubation/reintubation in adult critically ill patients.

**Results:**

We identified 18 RCTs (*n* = 4251 patients) in pooled analyses. As a primary mode of support, HFNC treatment reduced the risk of treatment failure [Odds Ratio (OR) 0.65; 95% confidence interval (CI) 0.43–0.98; *p* = 0.04; I^2^ = 32%] but had no effect on preventing intubation (OR, 0.74; 95%CI 0.45–1.21; *p* = 0.23; I^2^ = 0%) compared to COT. When used after extubation, HFNC (vs. COT) treatment significantly decreased reintubation rate (OR 0.46; 95%CI 0.33–0.63; *p* < 0.00001; I^2^ = 30%) and extubation failure (OR 0.43; 95%CI 0.25–0.73; *p* = 0.002; I^2^ = 66%). Compared to NIV, HFNC significantly reduced intubation rate (OR 0.57; 95%CI 0.36–0.92; *p* = 0.02; I^2^ = 0%) when used as initial support, but did no favorably impact clinical outcomes post extubation in few trials.

**Conclusions:**

HFNC was superior to COT in reducing treatment failure when used as a primary support strategy and in reducing rates of extubation failure and reintubation when used after extubation. In few trials, HFNC reduced intubation rate compared to NIV when used as initial support but demonstrated no beneficial effects after extubation.

**Electronic supplementary material:**

The online version of this article (10.1186/s12931-018-0908-7) contains supplementary material, which is available to authorized users.

## Background

Acute respiratory failure (ARF) is one of the most common causes of intensive care unit (ICU) mortality [1–3]. Oxygen therapy is a main stay of treatment for patients with hypoxemic respiratory failure. Several devices can be used to administer conventional oxygen treatments (COT), including nasal cannula, simple face masks, Venturi masks, and high-concentration reservoir masks [4]. The maximal flow rate that can be achieved with COT is 15 L/min which is lower than the inspiratory flow of most patients with ARF. Room air is often added to increase flow but at the expense of reducing the final concentration of oxygen delivered to patients at the alveolar level [5, 6]. Additionally, insufficient moisture and a lack of warm air during COT can induce discomfort for patients who require supplemental oxygen [7, 8].

High-flow nasal cannula (HFNC) delivers heated and humidified oxygen gas through the nasal or tracheal route with flow rates as high as 60 L/min in adults [6]. Several clinical trials have reported that HFNC improves oxygenation prior to intubation and reduces episodes of severe hypoxemia during intubation [9], post-cardiothoracic surgery [10], during bronchoscopy [11] and after extubation from invasive mechanical ventilation (IMV) in patients with ARF [12, 13]. Despite encouraging results from preliminary randomized controlled trials (RCTs), clarity is lacking regarding specific patient populations who may benefit from HFNC use [14–17]. To address this deficiency in the literature, we performed the current meta-analysis to compare the effect of HFNC, COT and noninvasive ventilation (NIV) on clinical outcomes of patients receiving either initial ARF treatment or respiratory support after extubation.

## Methods

### Inclusion and exclusion criteria

We included prospective RCTs involving adult patients comparing HFNC with either COT or NIV as an initial support strategy in patients with ARF or after extubation. We limited publications to adult patients (using author’s definitions) and the English language. We excluded crossover trials, before-after studies, abstract publications, conference presentations, case reports, editorials, and trials that included fewer than 20 patients in either treatment arm.

### Search strategy

To increase the sensitivity of our search strategy, we combined the terms “high flow oxygen” with “noninvasive ventilation” or “oxygen inhalation therapy” as key words or Medical Subject Headings (MeSH) terms. We searched 4 databases (Pubmed, EMBASE, Scopus, and Web of Science) from electronic databases inception to September, 1st, 2018. We systematically screened abstracts and full text publications for studies that met our eligibility criteria.

### Definitions

ARF was defined as the requirement for oxygen therapy to maintain peripheral capillary oxygen saturation (SpO_2_) > 92% or PaO_2_/FiO_2_ (P/F ratio) > 300, symptoms of respiratory distress (including tachypnea > 22 breaths/min, labored breathing, use of intercostal muscles, and/or dyspnea at rest) or using ‘authors’ definitions. HFNC was defined as respiratory support that delivered a high flow (> 15 L/min) of heated and humidified oxygen (37 °C) administered through nasal cannula. COT was referred to relatively low flow oxygen (≤ 15 L/min) through nasal cannula, a simple face mask, a Venturi mask, or a high-concentration reservoir mask. NIV included bilevel positive airway pressure and continuous positive airway pressure (CPAP). Treatment failure was defined as switching to a higher level respiratory support, (e.g., from HFNC or COT to NIV or IMV, or from HFNC or NIV to IMV). Extubation failure was defined as the need for NIV or reintubation within 72 h after HFNC use.

### Outcomes

The primary outcomes of this review were treatment failure and intubation (alternatively, reintubation rate in trials comparing alternative treatments after extubation) reflecting the efficacy of HFNC therapy (i.e., HFNC vs. COT, HFNC vs. NIV). Secondary outcomes included ICU and hospital mortality, ICU and hospital length of stay (LOS), patient comfort, respiratory rate (RR), and P/F ratio.

The four main comparisons in our review include (a) HFNC versus COT as initial support for patients with ARF; (b) HFNC versus COT to prevent extubation failure; (c) HFNC versus NIV in patients with ARF and (d) HFNC versus NIV after extubation. In a pre-specified subgroup analysis, we sought to compare the effect on intubation rate of HFNC vs. NIV in studies involving patients with severe hypoxemia (P/F ratio < 200 mmHg).

### Data abstraction

Three investigators (ZX, XL and JZ), working in pairs, independently reviewed and abstracted data from each retrieved article and supplement, where indicated. Discrepancies were resolved by discussion and consensus.

### Quality assessment

We assessed the quality of all included trials based on review of published trial protocols identified on trial registration sites ID (ClinicalTrials.gov; Australia New Zealand Clinical Trials Registry, Thai Clinical Trials Registry, International Standard Randomized Controlled Trial Number Registry) and the details in the method section and supplements of included trials. We appraised trial quality using the Cochrane collaboration tool for assessing risk of bias (RoB) [18] including assessment of random sequence generation, allocation concealment, blinding (of interventions and outcome measurement or assessment), incomplete outcome data, selective reporting bias and other potential sources of bias (e.g., industry funding). For each criterion, we appraised the RoB to be either of low, high, or unclear risk (e.g., insufficient details). Three authors (ZX, JZ, XL), working in pairs, independently assessed study quality and disagreements were resolved by consensus.

### Assessment of heterogeneity

We used the I^2^ statistic to evaluate the impact of heterogeneity on pooled results. An I^2^ value of greater than 50% indicated substantial heterogeneity [18]. We used fixed-effects models to pool data when heterogeneity was insignificant and the random effects models to pool data when significant heterogeneity was identified.

### Statistical analysis

Categorical data and continuous data were pooled using the odds ratios (ORs) and mean difference (MD), with the 95% confidence intervals (CIs). The Grading of Recommendation, Assessment, Development and Evaluation (GRADE) criteria were used to assess the quality of the evidence for HFNC on rates of intubation/reintubation since GRADE assigns high, moderate, low and very low classification based on assessment of study limitations, inconsistency, indirectness, imprecision, and publication bias [19]. Statistical analyses were conducted with Review Manager (RevMan) Version 5.3 (Copenhagen: The Nordic Cochrane Centre, The Cochrane Collaboration, 2014), and two-sided *p* values < 0.05 were considered statistically significant.

## Results

### Description of studies

**Fig. 1:**
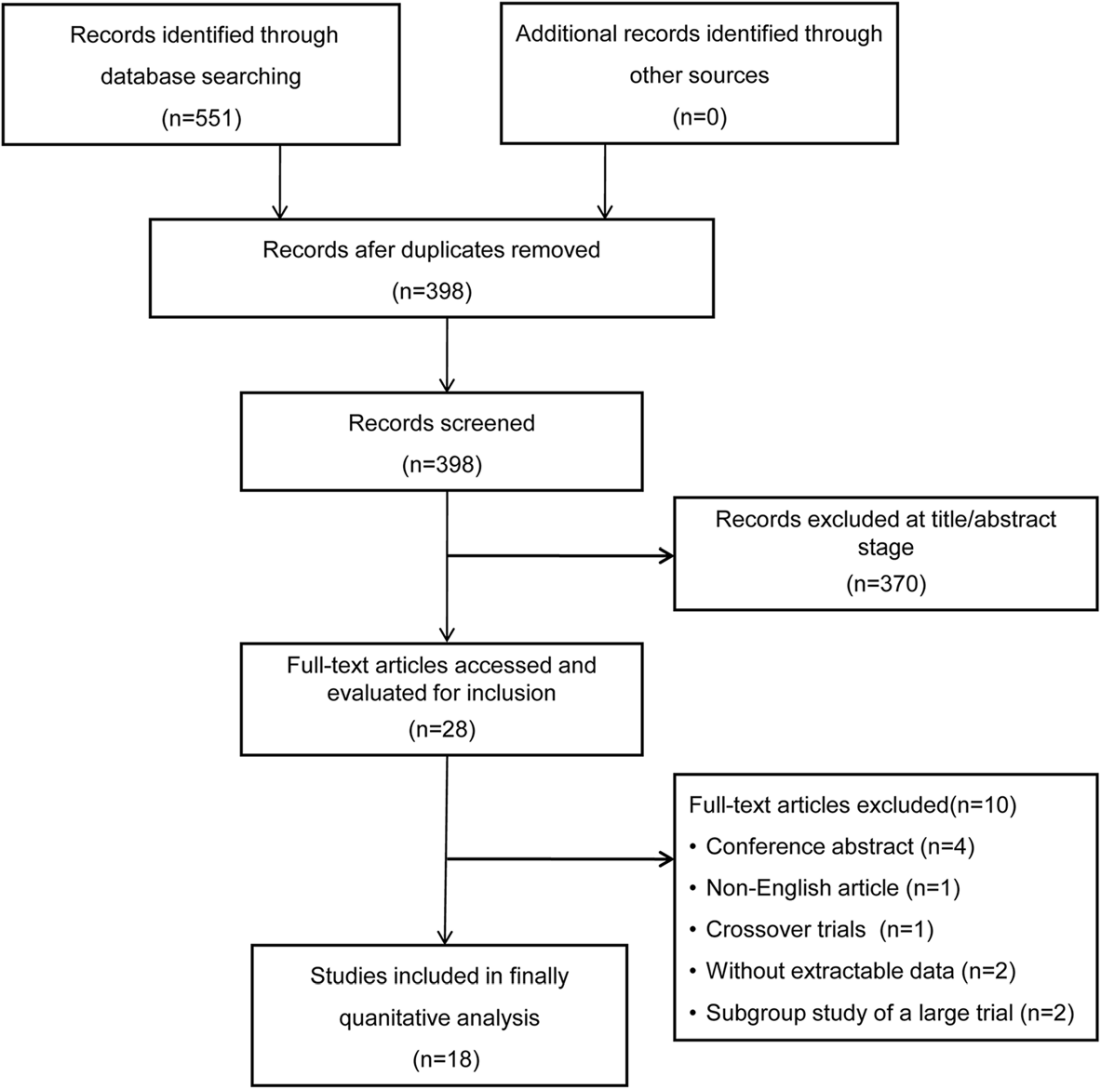
Search strategy of meta-analysis on selecting patients for inclusion

We identified 551 potentially eligible studies. After exclusion of duplicate and irrelevant articles, 28 trials were retrieved to be reviewed in greater detail. Of these, we excluded 10 studies that did not meet our eligibility criteria and thus included 18 trials in our review (Fig. 1, Additional files 1 and 2: Table S1). Of the 18 RCTs (*n* = 4251 including 2129 HFNC treated patients), 6 trials (*n* = 871) compared HFNC to COT [20–25] and 2 trials (*n* = 420) compared HFNC to NIV [24, 26] as an initial support strategy. For post-extubation use, 9 trials (*n* = 1731) compared HFNC to COT [12, 13, 27–33] and 2 trials (*n* = 1434) compared HFNC to NIV [10, 34]. Of these, 1 trial [24] compared HFNC, COT, and NIV treatment and data from this post-extubation trial were included in comparisons of HFNC vs. COT and HFNC vs. NIV. Two trials [25, 33] reported neither treatment failure nor intubation rate but included other secondary outcomes of interest.

### Risk of bias of included studies

**Fig. 2:**
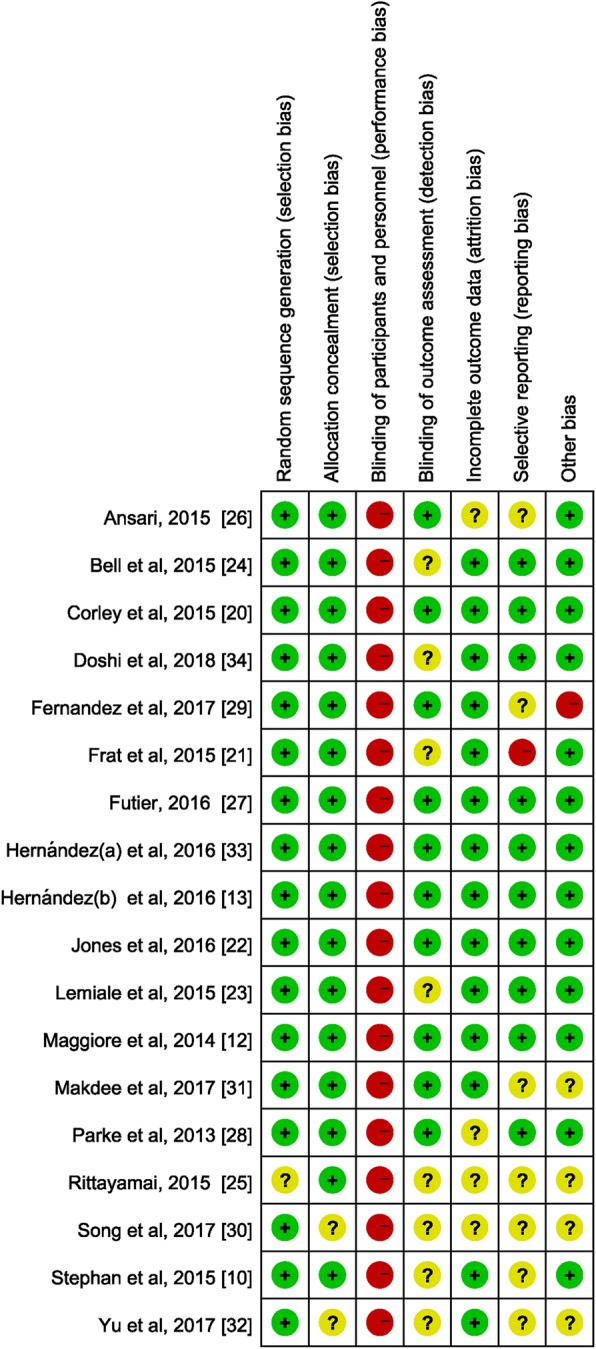
Risk of bias diagram for each study. Green represents low risk of bias, yellow represents unclear risk of bias, and red represents high risk of bias

The RCTs included were all assessed to be at low risk of bias with respect to randomization and allocation concealment except for 3 trials [25, 31, 32] for which selection bias was deemed unclear. The same 3 trials also were assessed to be at unclear risk of bias with regard to blinded outcome assessment, completeness of outcomes data, selective outcomes reporting, and other potential sources of bias [25, 31, 32]. All trials were deemed to be at high risk of performance bias as blinding of patients, physicians, and research personnel to treatment allocation was not feasible (Fig. 2).

### Primary outcomes

#### Trials comparing HFNC versus COT

##### HFNC versus COT as an initial support strategy

**Fig. 3:**
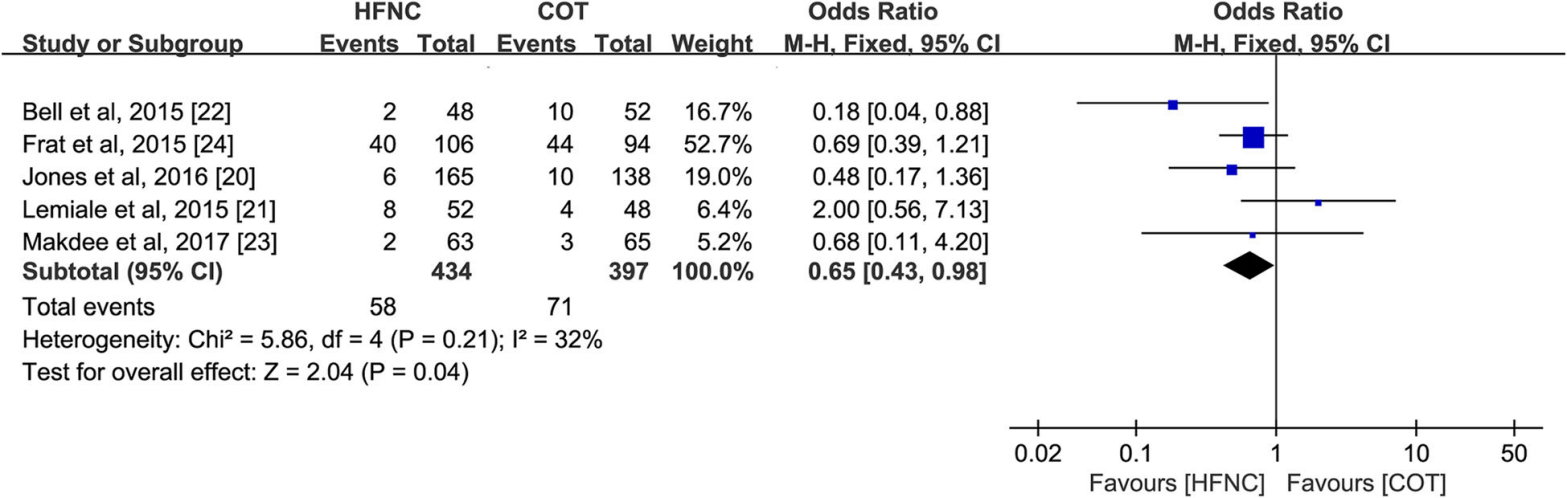
Treatment failure of HFNC versus COT as an initial support in ARF. Pooled estimates of treatment failure of HFNC compared with COT in patients used as an initial support

Five of 6 trials (*n* = 831) comparing HFNC and COT as an initial support strategy reported intubation and treatment failure rates [20–24]. Although HFNC had no effect on intubation (OR 0.74; 95%CI 0.45–1.21; *p* = 0.23; I^2^ = 0%), HFNC significantly reduced treatment failure (OR 0.65; 95%CI 0.43–0.98; *p* = 0.04; I^2^ = 32%) (Fig. 3).

##### HFNC versus COT after Extubation

**Fig. 4:**
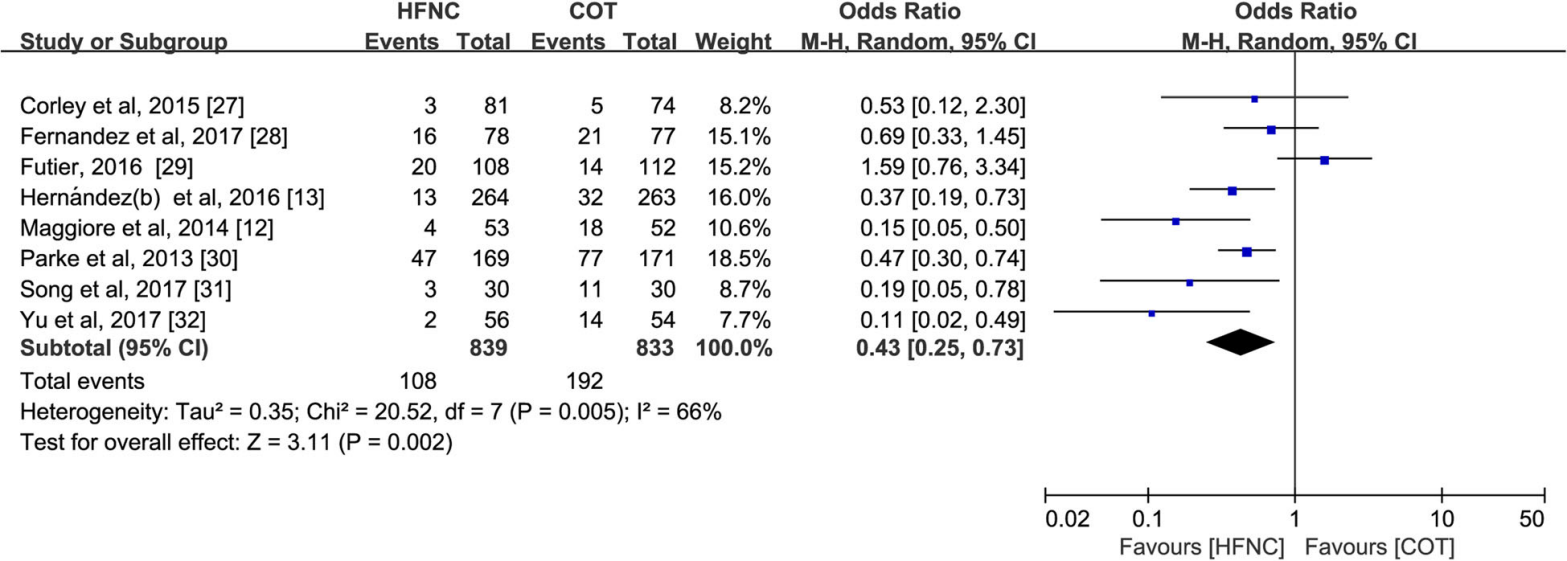
Treatment failure of HFNC versus COT after extubation. Pooled estimates of treatment failure of HFNC compared with COT in extubated patients from IMV

**Fig. 5:**
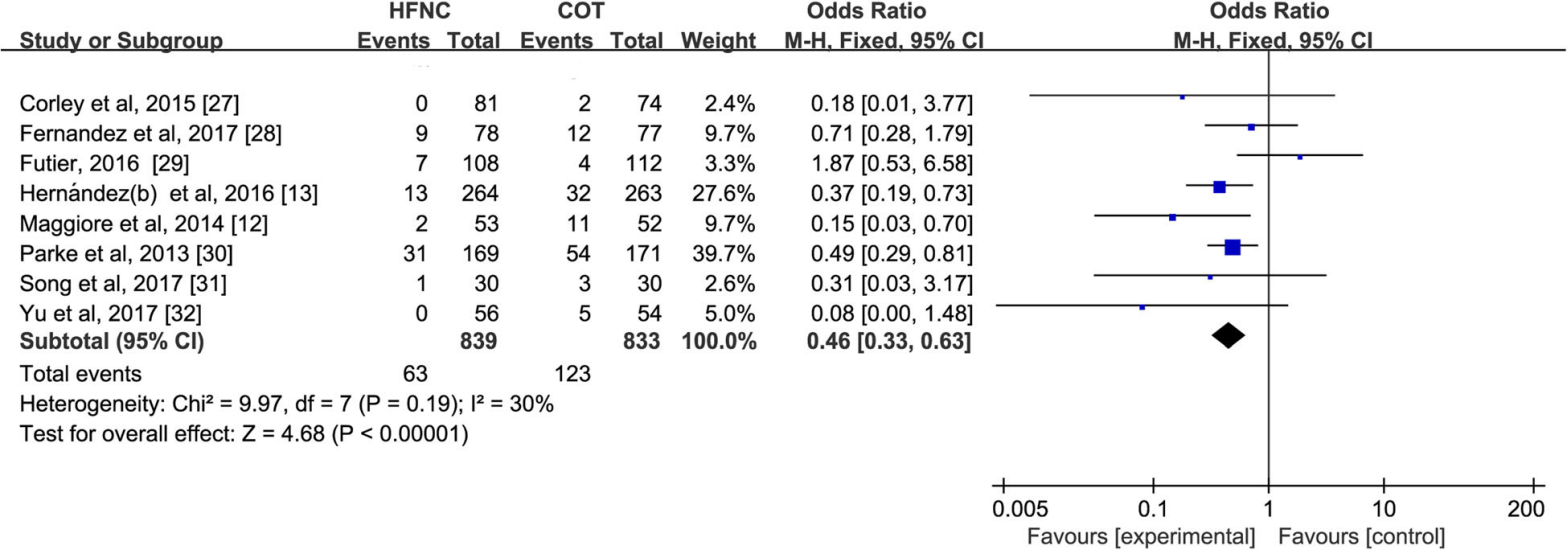
Reintubation rate of HFNC versus COT after extubation. Pooled estimates of risk of reintubation in patients after extubation supported on HFNC compared with COT

Eight of 9 trials (*n* = 1672) comparing HFNC with COT reported the effects of the alternative support strategies on rates of extubation failure and reintubation [12, 27–33]. Compared to COT, HFNC significantly reduced the risk of extubation failure (OR 0.43; 95%CI 0.25–0.73; *p* = 0.002; I^2^ = 66%) (Fig. 4) and reintubation (OR 0.46; 95%CI 0.33–0.63; *p* < 0.00001; I^2^ = 30%) (Fig. 5).

#### Trials comparing HFNC versus NIV

##### HFNC versus NIV as an initial support strategy

We pooled 2 trials (*n* = 420) that compared HFNC to NIV as an initial support strategy [24, 26]. Although HFNC had no effect on the rate treatment failure (OR 1.00; 95%CI 0.36–2.76; *p* = 1.00; I^2^ = 82%), it significantly reduced intubation rate in patients with ARF (OR 0.57; 95%CI 0.36–0.92; *p* = 0.02; I^2^ = 0%) (Additional file 2: Figure S1A).

##### HFNC versus NIV after Extubation

In 2 trials (*n* = 1434) comparing the effects of HFNC and NIV after extubation [10, 34], there was no significant difference in rates of treatment failure (OR 0.96; 95%CI 0.75–1.24; *p* = 0.77; I^2^ = 0%) and reintubation (OR 1.00; 95%CI 0.76–1.32; *p* = 0.98; I^2^ = 0%) (Additional file 2: Figure S1B).

### Secondary outcomes

#### Mortality and length of stay

We did not find differences in ICU and hospital mortality or lengths of stay when HFNC was compared to COT/NIV (Table 1).

**Table 1:** Secondary Outcomes

Clinical Outcome	No of Trials (number of patients)	Summary Estimate of Effect (Risk Ratio/Mean Difference with 95% CI)	*P*-value	I^2^ (%)
Trials Comparing HFNC vs. COT as an Initial Support Strategy				
ICU mortality	1(200)^a^	–	–	–
Hospital mortality	2(503)	0.72(0.42–1.25)	0.25	59%
ICU length of stay	–	–	–	–
Hospital length of stay	–	–	–	–
ED length of stay	3(531)^b^	–	–	–
Trials Comparing HFNC vs. COT After Extubation				
ICU mortality	3(787)	0.99(0.47–2.08)	0.97	0%
Hospital mortality	2(683)	0.87(0.47–1.58)	0.64	0%
ICU length of stay	4(710)	3.06(−0.56–6.69)	0.10	0%
Hospital length of stay	1(59)^a^	–	–	–
ED length of stay	–	–	–	–
Trials Comparing HFNC vs. NIV as an Initial Support Strategy				
ICU mortality	1(216)^a^	–	–	–
Hospital mortality	–	–	–	–
ICU length of stay	–	–	–	–
Hospital length of stay	–	–	–	–
ED length of stay	1(204)^a^	–	–	–
Trials Comparing HFNC vs. NIV After Extubation				
ICU mortality	2(1434)	1.20(0.87–1.85)	0.40	0%
Hospital mortality	–	–	–	–
ICU length of stay	1(604)^a^	–	–	–
Hospital length of stay	–	–	–	–
ED length of stay	–	–	–	–

*HFNC* High flow nasal cannulae, *COT* Conventional oxygen therapy, *ED* Emergency department

^a^only 1 trials was reported, no summary estimate of effect can be combined

^b^3 trials were included, but the data was expressed in different ways (mean/median), no summary estimate of effect can be combined

#### Patient comfort

Due to variability in reporting of scales used to assess comfort, we were unable to pool this data quantitatively. Qualitatively, 5 trials [12, 22–24, 31] found that HFNC was more comfortable than COT. Conversely, 3 trials [20, 25, 30] reported that COT was more comfortable than HFNC and 2 trials [21, 29] noted similar comfort ratings between HFNC and COT treated patients. In trials comparing HFNC and NIV, only 2 trials reported comfort scores with 1 study reporting greater comfort with HFNC [21] for patients with ARF and 1 trial [10] reporting similar comfort scores in patients after extubation (Additional file 2: Table S2).

#### Physiologic outcomes

We were unable to pool related to respiratory rate and P/F ratio due to variability in measuring and reporting these outcomes (Additional file 1). The results from trials were summarized in Additional file 2: Figures S3 and S4 and Tables S4 and S5.

### Subgroup analysis

In 2 trials including 640 patients with severe hypoxemia (P/F < 200 mmHg) [10, 24], HFNC had similar effects on intubation compared to NIV (OR 0.69; 95%CI 0.24–1.99; *p* = 0.49; I^2^ = 87%) (Additional file 2: Figure S2).

### Quality assessment

The strength of the evidence comparing HFNC to COT in ARF patients on treatment failure and intubation rate was of low quality, whereas for the comparison of HFNC with COT in extubation patients, the evidence on treatment failure and reintubation rate was of moderate quality. When comparing HFNC to NIV, both the intubation rate in ARF and reintubation rate in extubation patients were of low quality (Table 2).

**Table 2:** The GRADE Quality Assessment

Quality assessment							No of patients		Effect		Quality	Importance
No of studies	Design	Limitations	Inconsistency	Indirectness	Imprecision	Other considerations	HFNC	COT/NIV	Relative (95% CI)	Absolute		
Intubation rate of HFNC vs. COT as a primary mode												
5	randomised trials	serious^a^	no serious inconsistency	no serious indirectness	no serious imprecision	reporting bias^b^	46/434 (10.6%)	50/397 (12.6%)	OR 0.74 (0.45 to 1.21)	30 fewer per 1000 (from 65 fewer to 23 more)	⊕ ⊕ ΟΟ LOW	CRITICAL
Reintubation rate of HFNC vs. COT after extubation												
8	randomised trials	serious^a^	no serious inconsistency	no serious indirectness	no serious imprecision	reporting bias^b^ strong association^c^	63/839 (7.5%)	123/833 (14.8%)	OR 0.47 (0.29 to 0.76)	72 fewer per 1000 (from 31 fewer to 100 fewer)	⊕ ⊕ ⊕Ο MODERATE	CRITICAL
Intubation rate of HFNC vs. NIV as a primary mode												
2	randomised trials	serious^a^	no serious inconsistency	no serious indirectness	no serious imprecision	reporting bias^d^	47/210 (22.4%)	68/210 (32.4%)	OR 0.57 (0.36 to 0.92)	109 fewer per 1000 (from 18 fewer to 173 more)	⊕ ⊕ ΟΟ LOW	CRITICAL
Reintubation rate of HFNC vs. NIV after extubation												
2	randomised trials	serious^a^	no serious inconsistency	no serious indirectness	no serious imprecision	reporting bias^d^	118/704 (16.8%)	123/730 (16.8%)	OR 1.00 (0.76 to 1.32)	0 fewer per 1000 (from 35 fewer to 43 more)	⊕ ⊕ ΟΟ LOW	CRITICAL
Treatment failure of HFNC vs. COT as a primary mode												
5	randomised trials	serious^a^	no serious inconsistency	no serious indirectness	no serious imprecision	reporting bias^b^	58/434 (13.4%)	71/397 (17.9%)	OR 0.65 (0.43 to 0.98)	55 fewer per 1000 (from 3 fewer to 93 fewer)	⊕ ⊕ ΟΟ LOW	CRITICAL
Treatment failure of HFNC vs. COT after extubation												
8	randomised trials	serious^a^	serious inconsistency^e^	no serious indirectness	no serious imprecision	reporting bias^b^strong association^c^	108/893 (12.9%)	192/833 (23%)	OR 0.43 (0.25 to 0.73)	116 fewer per 1000 (from 51 fewer to 161 fewer)	⊕ ⊕ ⊕Ο MODERATE	CRITICAL

GRADE Working Group grades of evidence

High quality: Further research is very unlikely to change our confidence in the estimate of effect

Moderate quality: Further research is likely to have an important impact on our confidence in the estimate of effect and may change the estimate

Low quality: Further research is very likely to have an important impact on our confidence in the estimate of effect and is likely to change the estimate

Very low quality: We are very uncertain about the estimate

*CI* Confidence interval, *OR* Odds ratio

^a^Lack of blinding

^b^Funnel plot showed potential publication bias when HFNC vs. COT

^c^OR < 0.5

^d^Funnel plot showed potential publication bias when HFNC vs. NIV

^e^I^2^ = 66%

## Discussion

We found that HFNC was superior to COT in reducing treatment failure when used as an initial support strategy and reduced rates of extubation failure, and reintubation when used after extubation. In few trials, HFNC reduced intubation rate compared to NIV when used as initial support strategy but did not impact rates of treatment failure and reintubation when used after extubation.

To date meta-analyses have shown different effects of HFNC on intubation in patients with ARF [35–41]. The meta-analysis by Maitra et al. included 7 trials (*n* = 1699) found no benefit of HFNC compared to COT or NIV [35]. Subsequently, Monro-Somerville et al. combined data from 9 trials (*n* = 2507) comparing HFNC to other forms of respiratory support, including COT and NIV (as usual care), found no significant differences between treatment strategies in intubation and mortality rates [36]. Similarly, the review of Nedel and colleagues included 9 trials (*n* = 1552) of critically ill patients with or at risk of ARF found that HFNC therapy was not superior to COT or NIV [37]. In 2 trials (*n* = 495), a meta-analysis comparing HFNC in cardiac surgery patients, found that HFNC reduced escalation of respiratory support compared to COT [38]. Conversely, a recent meta-analysis by Ni et al. pooled 8 studies (*n* = 1084) including RCTs and retrospective studies and identified that HFNC reduced intubation rate compared to COT and NIV [39]. Huang et al. had found that HFNC may be benefit to avoid reintubation in critically ill patients with ARF by pooling data of 7 trials (*n* = 2781) [40]. The most recent review by Zhao et al. included 11 trials (*n* = 3459) compared HFNC to COT or NIV [41] and found that HFNC reduced intubation rate compared to COT but not to NIV. Our meta-analysis differs from previous meta-analyses in the inclusion criteria utilized, the number of trials and patients included, the outcomes reported, and in summary estimates of treatment effect. We focused on clinical indications for use of HFNC and compared its use to alternative treatments (COT and NIV). Our review represents the largest meta-analysis conducted to date including 18 RCTs and 4251 patients. We found that HFNC (vs. COT) reduced treatment failure when used as an initial support strategy in patients with ARF. Contrary to the findings of Zhao et al. [41], we found that compared to COT, HFNC reduced the rate of treatment failure (low quality) but not intubation rate (low quality evidence). Additionally, we found that HFNC (vs. COT) significantly reduced rates of both extubation failure (moderate quality evidence) and reintubation (moderate quality evidence) when used after extubation. Similar to studies conducted preterm infants, these findings suggests a potential clinical role for HFNC in the post extubation period [42]. Finally, compared to NIV, we found promising preliminary data in 2 trials that HFNC may reduce the rate of intubation when used as an initial support strategy. Taken together these findings support the use of HFNC versus COT as an initial support strategy and after extubation. Notwithstanding, several questions regarding HFNC application remain to be addressed. Further trials are needed to clarify the role for HFNC in different etiologies of ARF and compared to NIV after extubation. Several trials are currently underway to evaluate the effect of HFNC in moderate and severe ARF and in AECOPD (ClinicalTrials.gov: NCT02687074, NCT02439333).

Although HFNC therapy was initially developed for neonatal patients, indications for its use have recently been expanded to include adult patients [6, 43]. Several mechanisms have been postulated to improve oxygenation in patients who are treated with HFNC. First, the high flow rates with HFNC ‘washout’ carbon dioxide in upper airways and reduce dead space [44]. Second, the peak inspiratory flow of dyspneic patients can be met, and even exceeded, by the administration of high flow gas with HFNC thus reducing the dilution effects of the administered gas with room air [5]. Third, the ability to heated and humidified gas with HFNC facilitates tolerance [45]. Fourth, HFNC may create a small amount of positive pressure in the nasopharynx [46], which may help prevent atelectasis and recruit collapsed alveoli [47]. Patients, especially those with hypoxemic ARF, may benefit from some or all of the purported mechanisms of action associated with HFNC.

Our meta-analysis has several strengths. It is the largest meta-analyses conducted to date and to evaluate HFNC use by clinical indication. It is strengthened by an extensive search, duplicate citation screening and data abstraction, and conduct of a prespecified subgroup analysis. Our meta-analysis also has limitations. First, despite an extensive literature search, we identified only 4 trials [10, 24, 26, 34] comparing HFNC and NIV including 2 initial support strategy trials and 2 trials post-extubation trials. Second, by necessity all trials were deemed to be at high risk of performance bias as the nature of the interventions being applied precluded blinding after treatment allocation. Third, we did not construct funnel plots as fewer than 10 trials were identified for each comparison. Finally, we were not able to pool all data reported for outcomes including ICU and hospital stay, respiratory rate, and PaO_2_/FiO_2_ ratio due to variability in measuring and reporting of these outcomes.

## Conclusions

We found that compared to COT, HFNC significantly reduced treatment failure when used as an initial support strategy and when used after extubation reduced both extubation failure and reintubation rates. In few trials, HFNC reduced intubation rate compared to NIV when used as initial support strategy but did not impact rates of treatment failure and reintubation when used after extubation.

## Additional files

Additional file 1: Description of Studies and other Secondary Outcomes. (DOC 133 kb)

Additional file 2: Additional Tables and Figures. (ZIP 5033 kb)

## Abbreviations

AECOPD: Acute exacerbation of chronic obstructive pulmonary disease
ARF: Acute respiratory failure
CI: Confidence interval
COT: Conventional oxygen therapy
CPAP: Continuous positive airway pressure
ED: Emergence department
GRADE: The grading of recommendation, assessment, development and evaluation
HFNC: High-flow nasal cannula
ICU: Intensive care unit
IMV: Invasive mechanical ventilation
LOS: Length of stay
MD: Mean difference
NIV: Noninvasive ventilation
OR: Odds ratio
P/F ratio: PaO_2_/FiO_2_ ratio
RCTs: Randomized controlled trials
Rob: Risk of bias
RR: Respiratory rate
