# Supplementary material for: High-flow nasal cannula in adults with acute respiratory failure and after extubation: a systematic review and meta-analysis
Source: Respir Res. 2018 Oct 16;19:202. doi: 10.1186/s12931-018-0908-7 (PMC6192218; doi:10.1186/s12931-018-0908-7)
Supplement: Supplementary file 1 — Description of Studies and other Secondary Outcomes. (DOC 133 kb) [file 12931_2018_908_MOESM1_ESM.doc]

**High-flow nasal cannula in adults with acute respiratory failure and**

**After extubation: A Systematic Review and Meta-Analysis**

Zhiheng Xu^1,2*^, Yimin Li^1,2*^, Jianmeng Zhou^1^, Xi Li^1,2^, Yongbo Huang^1,2^, Xiaoqing Liu^1^,Karen E. A.Burns^3,4^,Nanshan Zhong^1^,Haibo Zhang^1-4^

^1^State Key Laboratory of Respiratory Diseases, National Clinical Research Center for Respiratory Disease, Guangzhou Institute for Respiratory Health, Guangzhou, China

^2^Department of Critical Care Medicine, The First Affiliated Hospital of Guangzhou Medical University, Guangzhou, China

^3^Interdepartmental Division of Critical Care Medicine, University of Toronto, Ontario, Canada

^4^The Keenan Research Centre for Biomedical Science of St.Michael's Hospital and Departments of Anesthesia and Physiology, University of Toronto, Toronto, Ontario, Canada

*Co-first authors

Supplementary appendix

**Description of Studies**

We identified 551 references. After exclusion of duplicate or irrelevant references, 28 potential articles were reviewed in detail. Of these, 4 studies were published as conference abstracts and a crossover trial and a non-English article were excluded. Additionally, we excluded a post-hoc subgroup analysis of the FLORALI study due to duplicated patients. We excluded a post-hoc subgroup analysis of the GRRR-OH trial due to the use of HFNC at clinician’s discretion without randomization. And the study by Parke et al comparing high flow oxygen therapy with face mask or nasal cannula . In addition, we excluded the study from Brainard et al due to high risks of bias of retrospectively registered . Although the patients in the phase II study OPERA study of HFNC did not have ARF based on baseline characteristics , they were included in post-extubation comparisons. The 2 studies by Rittayamai et al and Ansari et al , did not report data on treatment failure or intubation rate, however, they were included as they reported secondary outcomes of interest. The FLORALI study (8) included data comparing HFNC, COT, and NIV treatments, and contributed to both analyses of HFNC versus COT, and HFNC versus NIV. We included 18 randomized control trials in the present meta-analysis.

**Secondary outcome**

**Respiratory Rate**

Seven trials reported the effect HFNC compared with COT on respiratory rate (RR). Compared with COT as a primary support strategy, HFNC in 3 trials (n=368) significantly reduced RR (MD -3.23; 95%CI -4.28 to -2.18; p<0.00001) in the first hour without heterogeneity (I^2^ = 0%). There were 6 trials (n=926) reporting similar RR in the later time point when HFNC was compared with COT except one trial. Compared to NIV, HFNC had no effect in reducing RR at the first hour (MD -1.21; 95%CI -4.63 to 2.22; p=0.49; I^2^=86%) (Fig. S3 & Tables S4).

**PaO_2_/FiO_2_ Ratio**

Three trials showed no improvement of PaO_2_/FiO_2_ ratio at 24hours (MD 16.41; 95%CI -7.60 to 40.43; p=0.18) with HFNC vs. COT in patients after extubation amidst significant heterogeneity (p=0.0004; I^2^=87%). The sensitivity analysis showed that heterogeneity could be attributed to 1 trial. When this trial was excluded, HFNC significantly improved oxygenation at 24h (MD 26.05; 95%CI 10.89 to 41.21; p=0.0008) when compared with COT in patients after extubation with less heterogeneity (p=0.29; I^2^=12%). However, NIV significantly improved oxygenation when compared to HFNC except in one study (Fig. S4 & Table S5).

**Figure S1:** Trials comparing HFNC versus NIV

**Figure S2:** Needing intubation of HFNC versus NIV in patients with P/F<200mmHg **Figure S3:** Respiratory rate (RR) (mean [SD] or median [IQR])) breaths/min

**Figure S4:** PaO_2_/FiO_2_ ratio of HFNC vs. COT in patients after extubation

**Table S1:** Characteristics of included studies

**Table S2:** Comfort/discomfort in patients received HFNC/COT/NIV

**Table S3:** Length of stay in ED/ICU/hospital (mean [SD] or median [IQR]))

**Table S4:** Respiratory rate (RR) (mean [SD] or median [IQR])) breaths/min

**Table S5:** PaO_2_/FiO_2_ ratio (mean [SD] or median [IQR]) mmHg

Reference:

1. Cracchiolo AN, Palma DM, Tetamo R. High flow nasal cannula vs standard oxygen face mask during physiotherapy in brain injury patients: A feasible study. Intensive Care Medicine Experimental. 2017;5(2).

2. Sharpe AL, Mathews KS, Ranginwala S, Richman LS. Outcomes associated with non-invasive management of pneumonia-related acute hypoxemic respiratory failure. American Journal of Respiratory and Critical Care Medicine. 2017;195.

3. Theerawit P, Natpobsuk N, Sutherasan Y. The efficacy of the Whispherflow CPAP system versus high flow nasal cannula in patients at high risk for postextubation failure. Intensive Care Medicine Experimental. 2017;5(2).

4. Wiboonsirichai N, Kongpolprom N, Desudchit T, Sittipunt C. Effects of high flow oxygen therapy on oxygen desaturation index in patients with acute ischemic stroke. Respirology. 2017;22:38.

5. Hui D, Morgado M, Chisholm G, Withers L, Nguyen Q, Finch C, et al. High-Flow Oxygen and Bilevel Positive Airway Pressure for Persistent Dyspnea in Patients With Advanced Cancer: A Phase II Randomized Trial. Journal Of Pain And Symptom Management. 2013;46(4):463-73.

6. Nicolet J, Poulard F, Baneton D, Rigal JC, Blanloeil Y. [High-flow nasal oxygen for severe hypoxemia after cardiac surgery]. Annales francaises d'anesthesie et de reanimation. 2011;30(4):331-4.

7. Frat JP, Ragot S, Girault C, Perbet S, Prat G, Boulain T, et al. Effect of non-invasive oxygenation strategies in immunocompromised patients with severe acute respiratory failure: A post-hoc analysis of a randomised trial. The Lancet Respiratory Medicine. 2016.

8. Frat JP, Thille AW, Mercat A, Girault C, Ragot S, Perbet S, et al. High-flow oxygen through nasal cannula in acute hypoxemic respiratory failure. New England Journal of Medicine. 2015;372(23):2185-96.

9. Lemiale V, Resche-Rigon M, Mokart D, Pène F, Argaud L, Mayaux J, et al. High-Flow Nasal Cannula Oxygenation in Immunocompromised Patients With Acute Hypoxemic Respiratory Failure: A Groupe de Recherche Respiratoire en Réanimation Onco-Hématologique Study. Critical care medicine. 2016.

10. Lemiale V, Mokart D, Resche-Rigon M, Pene F, Mayaux J, Faucher E, et al. Effect of Noninvasive Ventilation vs Oxygen Therapy on Mortality Among Immunocompromised Patients With Acute Respiratory Failure: A Randomized Clinical Trial. JAMA. 2015;314(16):1711-9.

11. Parke RL, McGuinness SP, Eccleston ML. A Preliminary Randomized Controlled Trial to Assess Effectiveness of Nasal High-Flow Oxygen in Intensive Care Patients. Respiratory care. 2011;56(3):265-70.

12. Brainard J, Scott BK, Sullivan BL, Fernandez-Bustamante A, Piccoli JR, Gebbink MG, et al. Heated humidified high-flow nasal cannula oxygen after thoracic surgery A randomized prospective clinical pilot trial. Journal Of Critical Care. 2017;40:225-8.

13. Parke R, McGuinness S, Dixon R, Jull A. Open-label, phase II study of routine high-flow nasal oxygen therapy in cardiac surgical patients. British Journal of Anaesthesia. 2013;111(6):925-31.

14. Futier E, Paugam-Burtz C, Godet T, Khoy-Ear L, Rozencwajg S, Delay JM, et al. Effect of early postextubation high-flow nasal cannula vs conventional oxygen therapy on hypoxaemia in patients after major abdominal surgery: a French multicentre randomised controlled trial (OPERA). Intensive care medicine. 2016;42(12):1888-98.

15. Rittayamai N, Tscheikuna J, Praphruetkit N, Kijpinyochai S. Use of High-Flow Nasal Cannula for Acute Dyspnea and Hypoxemia in the Emergency Department. Respiratory care. 2015;60(10):1377-82.

16. Ansari BM, Hogan MP, Collier TJ, Baddeley RA, Scarci M, Coonar AS, et al. A Randomized Controlled Trial of High-Flow Nasal Oxygen (Optiflow) as Part of an Enhanced Recovery Program After Lung Resection Surgery. Annals of Thoracic Surgery. 2016;101(2):459-64.

17. Jones PG, Kamona S, Doran O, Sawtell F, Wilsher M. Randomized controlled trial of humidified high-flow nasal oxygen for acute respiratory distress in the emergency department: The HOT-ER study. Respiratory care. 2016;61(3):291-9.

18. Corley A, Bull T, Spooner AJ, Barnett AG, Fraser JF. Direct extubation onto high-flow nasal cannulae post-cardiac surgery versus standard treatment in patients with a BMI ≥30: a randomised controlled trial. Intensive care medicine. 2015;41(5):887-94.

19. Lemiale V, Mokart D, Mayaux J, Lambert J, Rabbat A, Demoule A, et al. The effects of a 2-h trial of high-flow oxygen by nasal cannula versus Venturi mask in immunocompromised patients with hypoxemic acute respiratory failure: A multicenter randomized trial. Critical Care. 2015;19(1).

20. Makdee O, Monsomboon A, Surabenjawong U, Praphruetkit N, Chaisirin W, Chakorn T, et al. High-Flow Nasal Cannula Versus Conventional Oxygen Therapy in Emergency Department Patients With Cardiogenic Pulmonary Edema: A Randomized Controlled Trial. Annals of emergency medicine. 2017;70(4):465-72.e2.

21. Song HZ, Gu JX, Xiu HQ, Cui W, Zhang GS. The value of high-flow nasal cannula oxygen therapy after extubation in patients with acute respiratory failure. Clinics (Sao Paulo, Brazil). 2017;72(9):562-7.

22. Hernandez G, Vaquero C, Gonzalez P, Subira C, Frutos-Vivar F, Rialp G, et al. Effect of Postextubation High-Flow Nasal Cannula vs Conventional Oxygen Therapy on Reintubation in Low-Risk Patients A Randomized Clinical Trial. Jama-Journal of the American Medical Association. 2016;315(13):1354-61.

23. Maggiore SM, Idone FA, Vaschetto R, Festa R, Cataldo A, Antonicelli F, et al. Nasal high-flow versus Venturi mask oxygen therapy after extubation. Effects on oxygenation, comfort, and clinical outcome. American journal of respiratory and critical care medicine. 2014;190(3):282-8.

24. Hernandez G, Vaquero C, Colinas L, Cuena R, Gonzalez P, Canabal A, et al. Effect of Postextubation High-Flow Nasal Cannula vs Noninvasive Ventilation on Reintubation and Postextubation Respiratory Failure in High-Risk Patients: A Randomized Clinical Trial. Jama. 2016.
