# Supplementary figures and images for: High-flow nasal cannula in adults with acute respiratory failure and after extubation: a systematic review and meta-analysis
Source: Respir Res. 2018 Oct 16;19:202. doi: 10.1186/s12931-018-0908-7 (PMC6192218; doi:10.1186/s12931-018-0908-7)

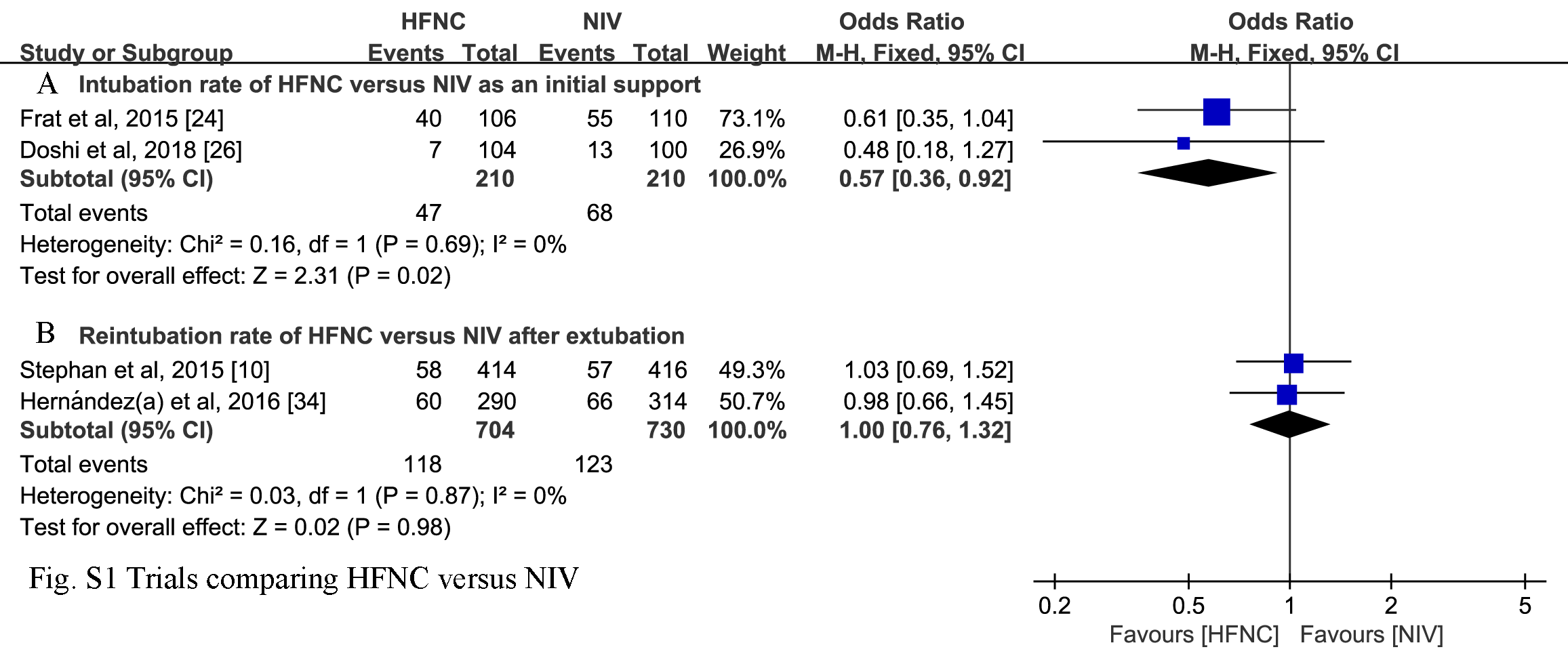

Supplement: Supplementary file 2 — Additional Tables and Figures. (ZIP 5033 kb) [file 12931_2018_908_MOESM2_ESM.zip › Fig. S1.tif]

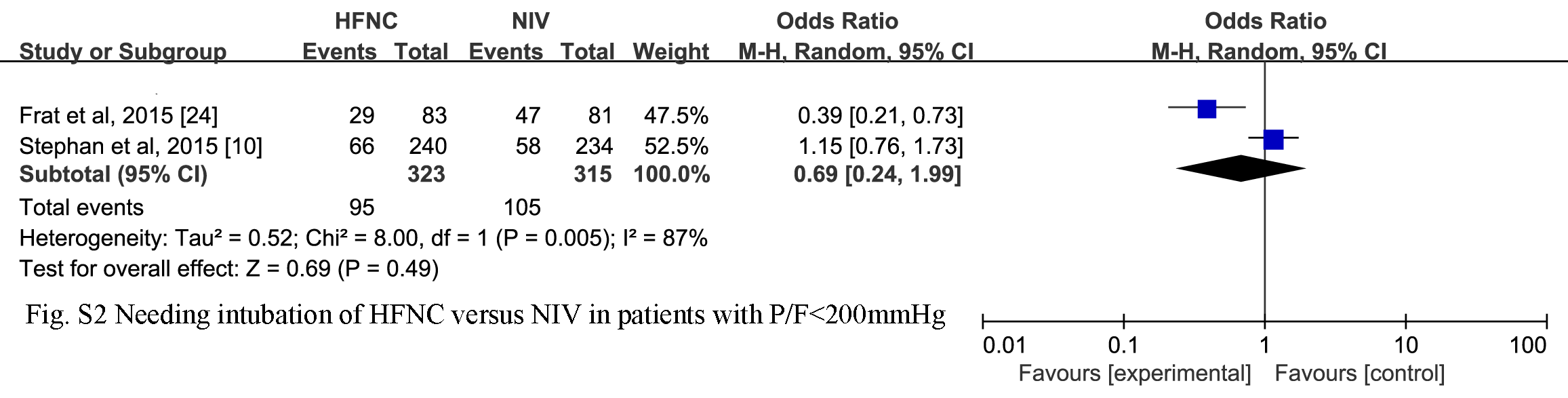

Supplement: Supplementary file 2 — Additional Tables and Figures. (ZIP 5033 kb) [file 12931_2018_908_MOESM2_ESM.zip › Fig. S2.tif]

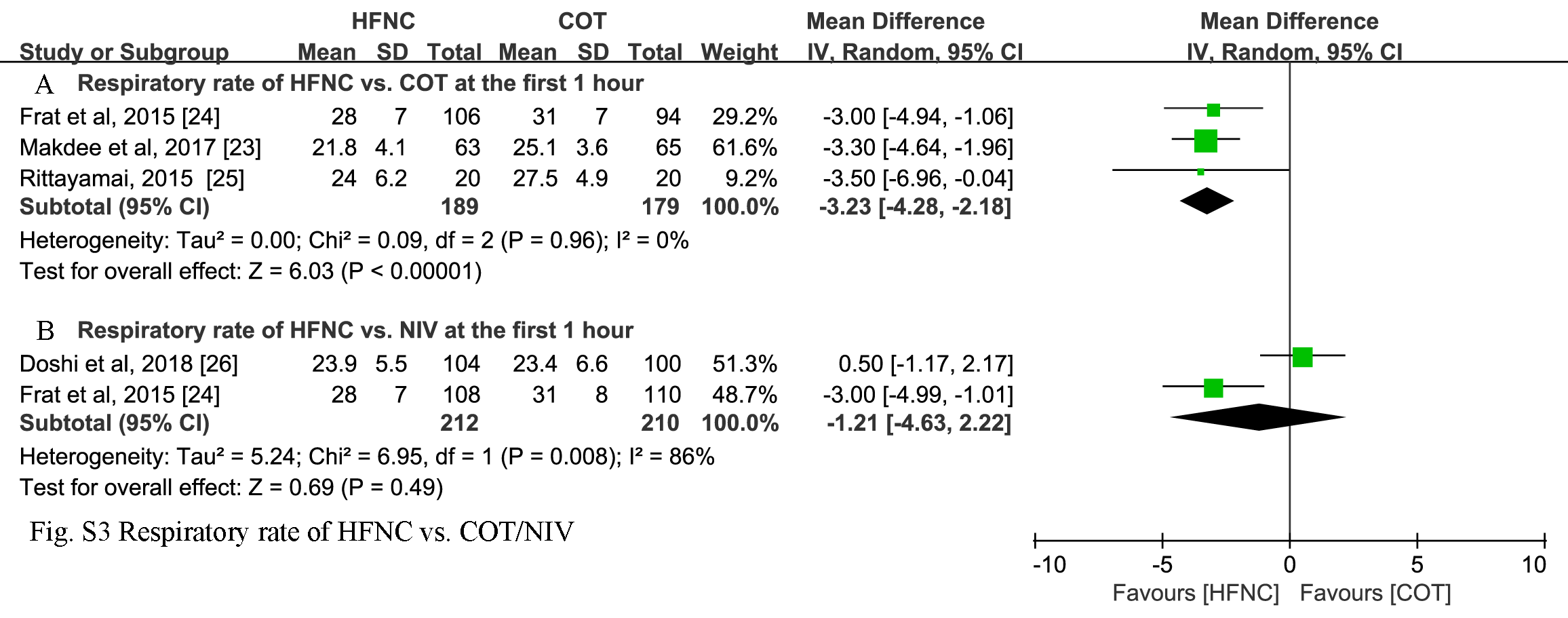

Supplement: Supplementary file 2 — Additional Tables and Figures. (ZIP 5033 kb) [file 12931_2018_908_MOESM2_ESM.zip › Fig.S3.tif]

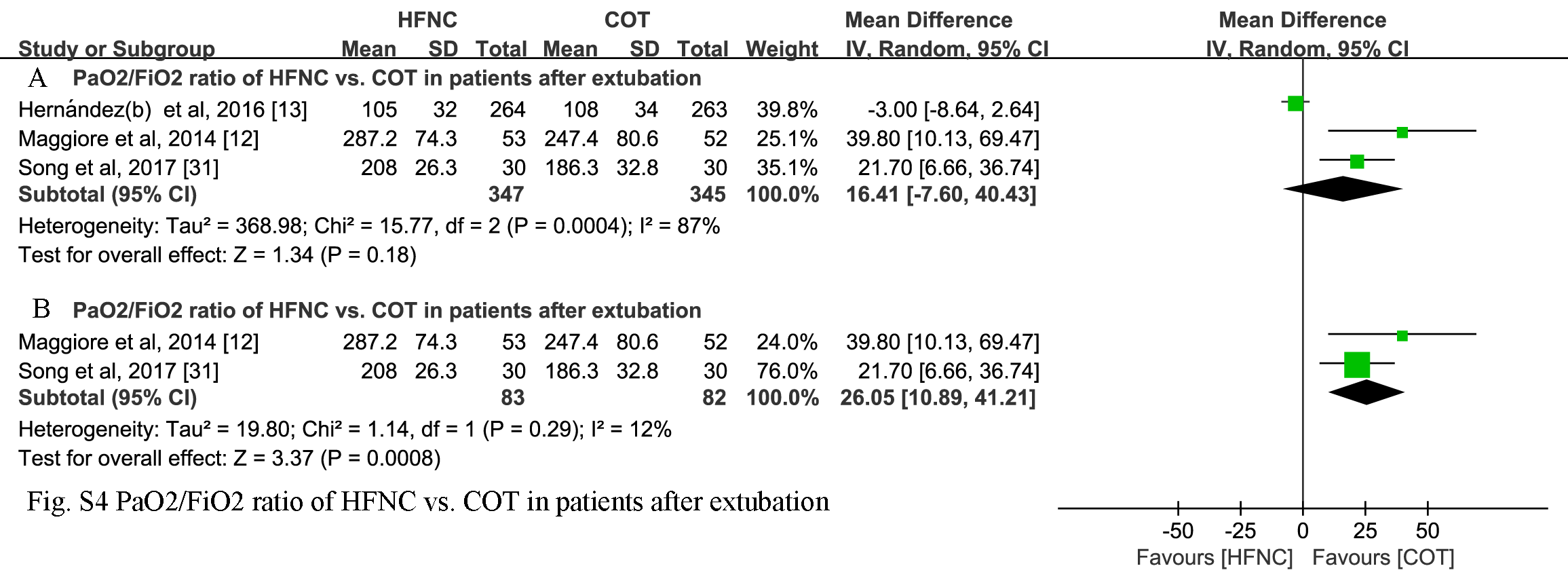

Supplement: Supplementary file 2 — Additional Tables and Figures. (ZIP 5033 kb) [file 12931_2018_908_MOESM2_ESM.zip › Fig.S4.tif]

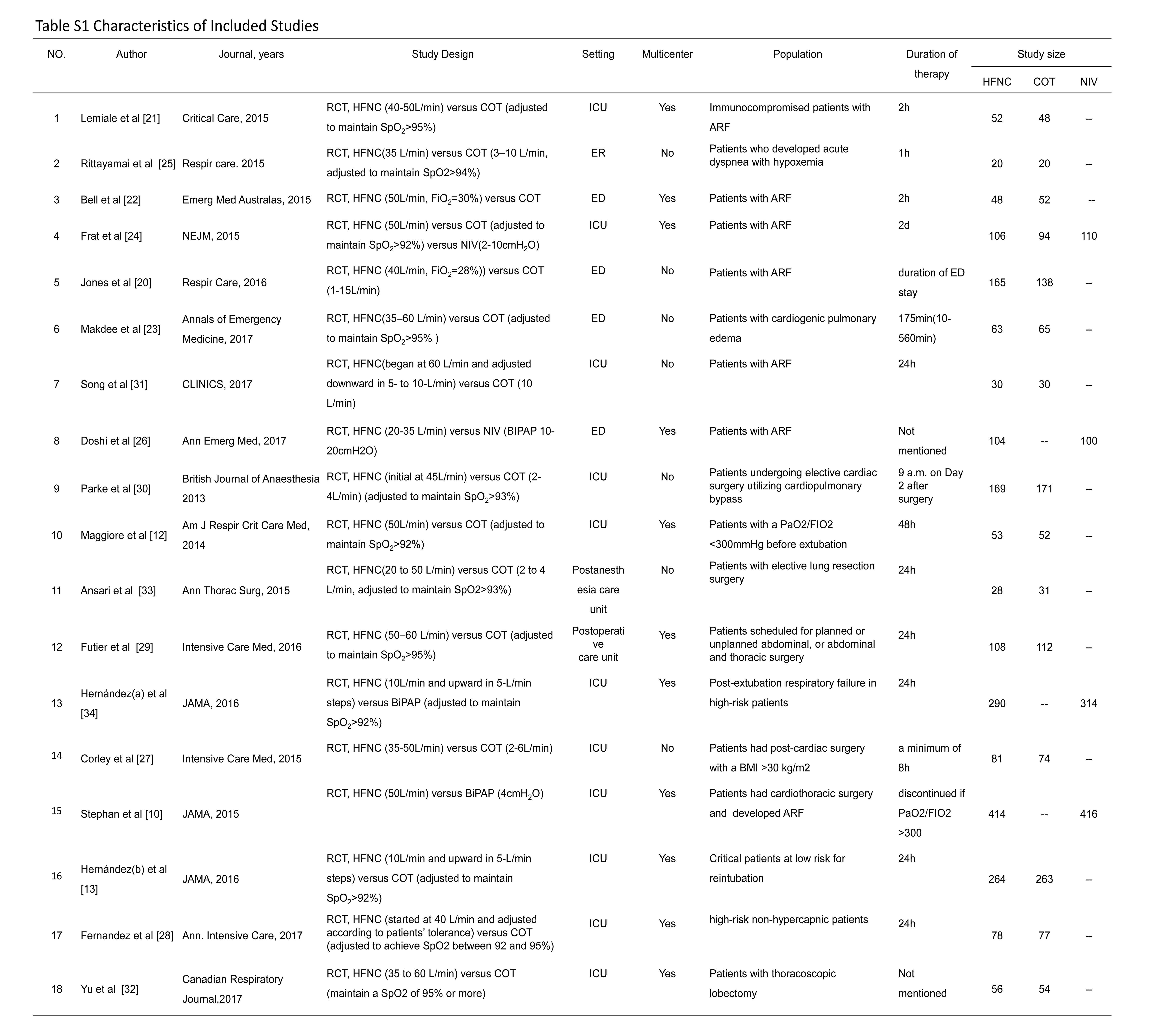

Supplement: Supplementary file 2 — Additional Tables and Figures. (ZIP 5033 kb) [file 12931_2018_908_MOESM2_ESM.zip › table S1 Characteristics of included studies.tif]

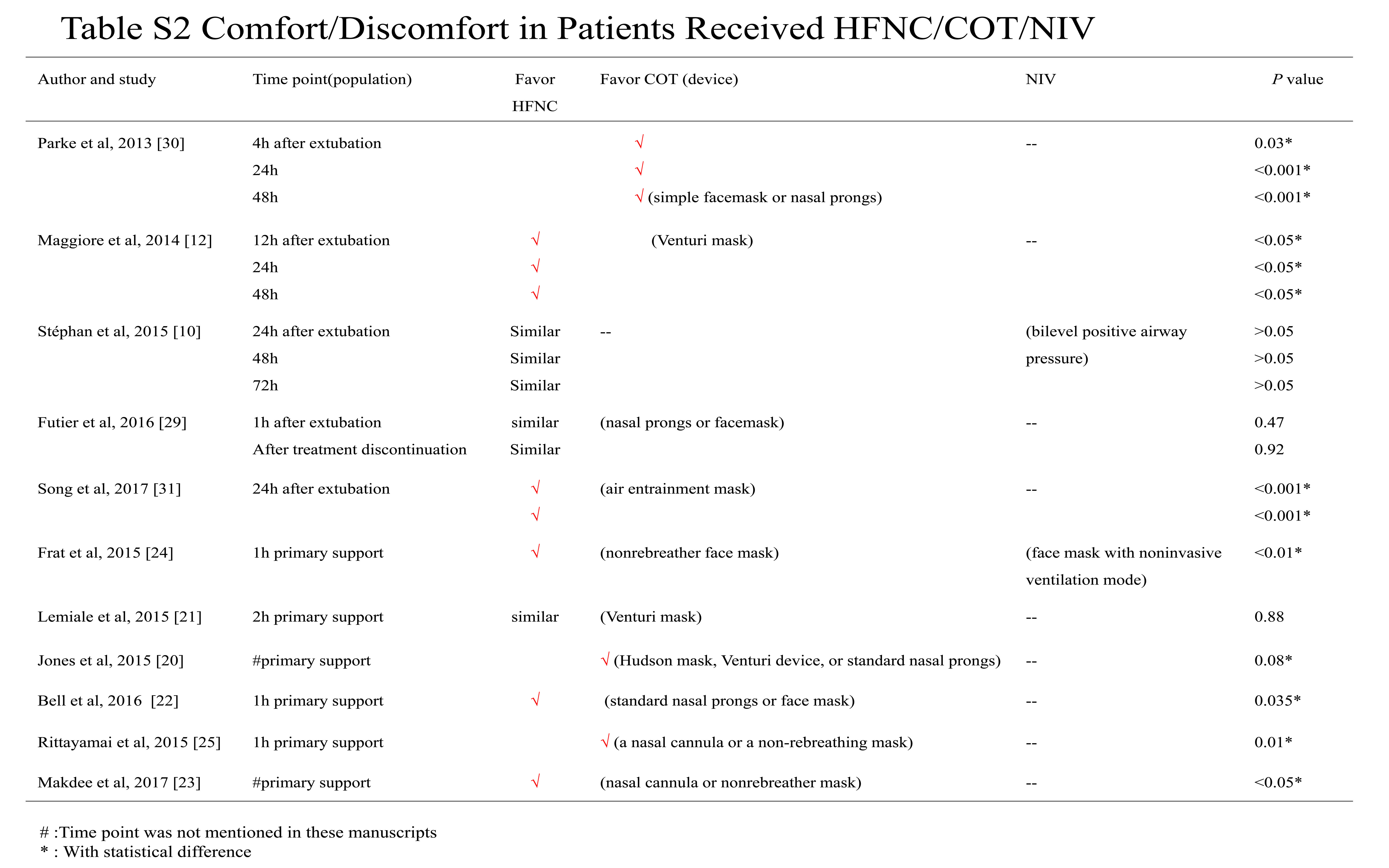

Supplement: Supplementary file 2 — Additional Tables and Figures. (ZIP 5033 kb) [file 12931_2018_908_MOESM2_ESM.zip › table S2 discomfort.tif]

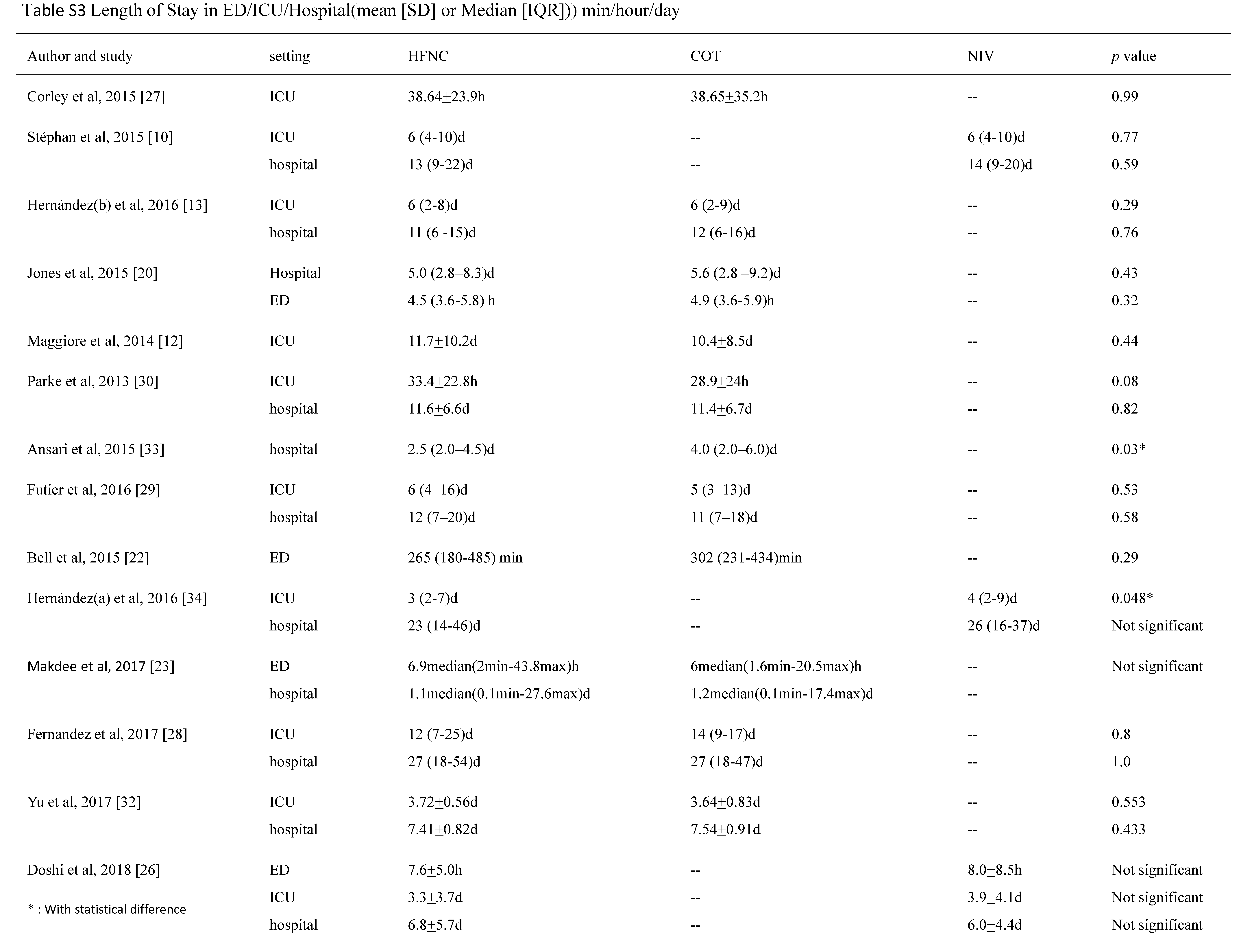

Supplement: Supplementary file 2 — Additional Tables and Figures. (ZIP 5033 kb) [file 12931_2018_908_MOESM2_ESM.zip › table S3 Length of stay.tif]

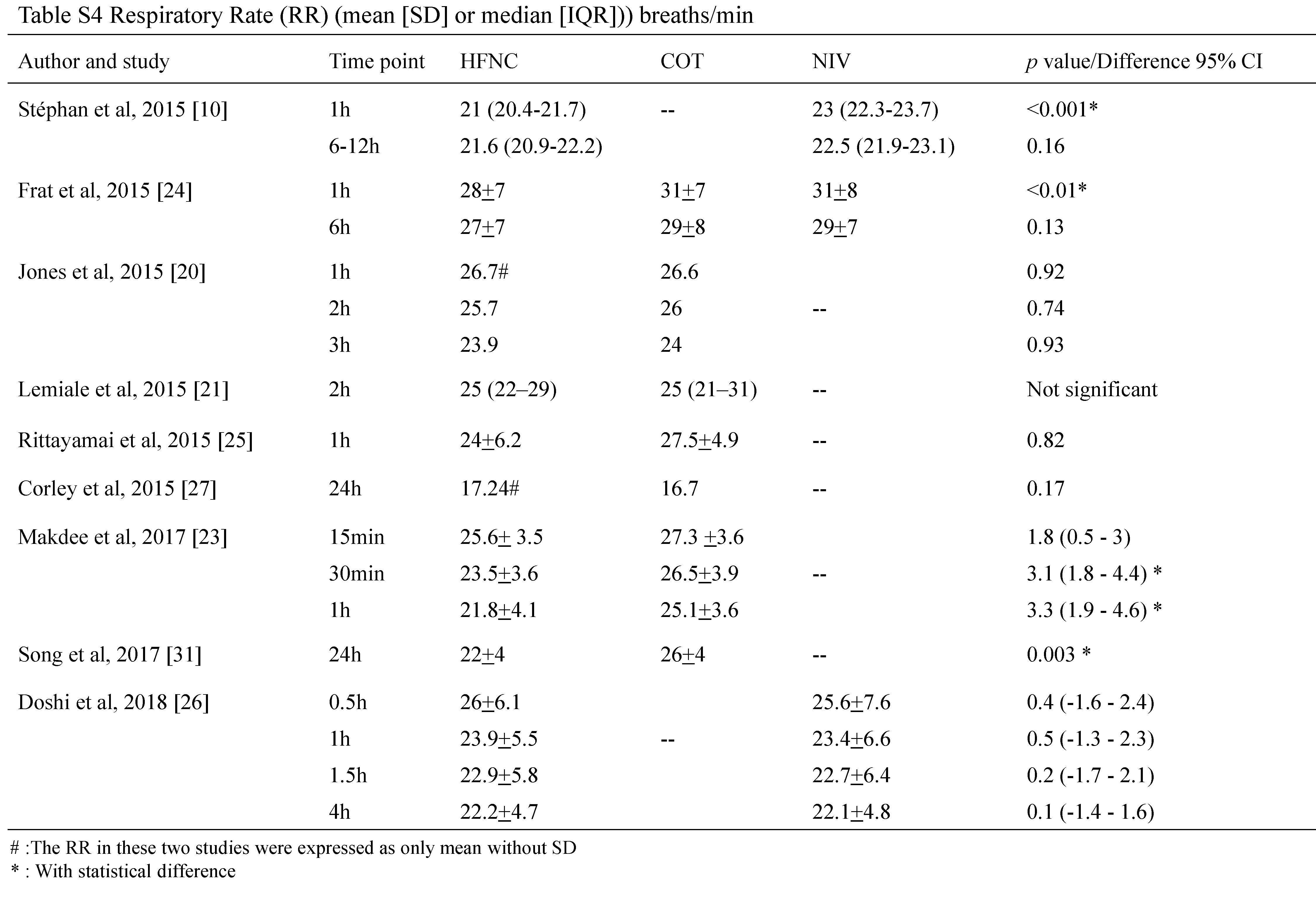

Supplement: Supplementary file 2 — Additional Tables and Figures. (ZIP 5033 kb) [file 12931_2018_908_MOESM2_ESM.zip › table S4 Respiratory rate.tif]

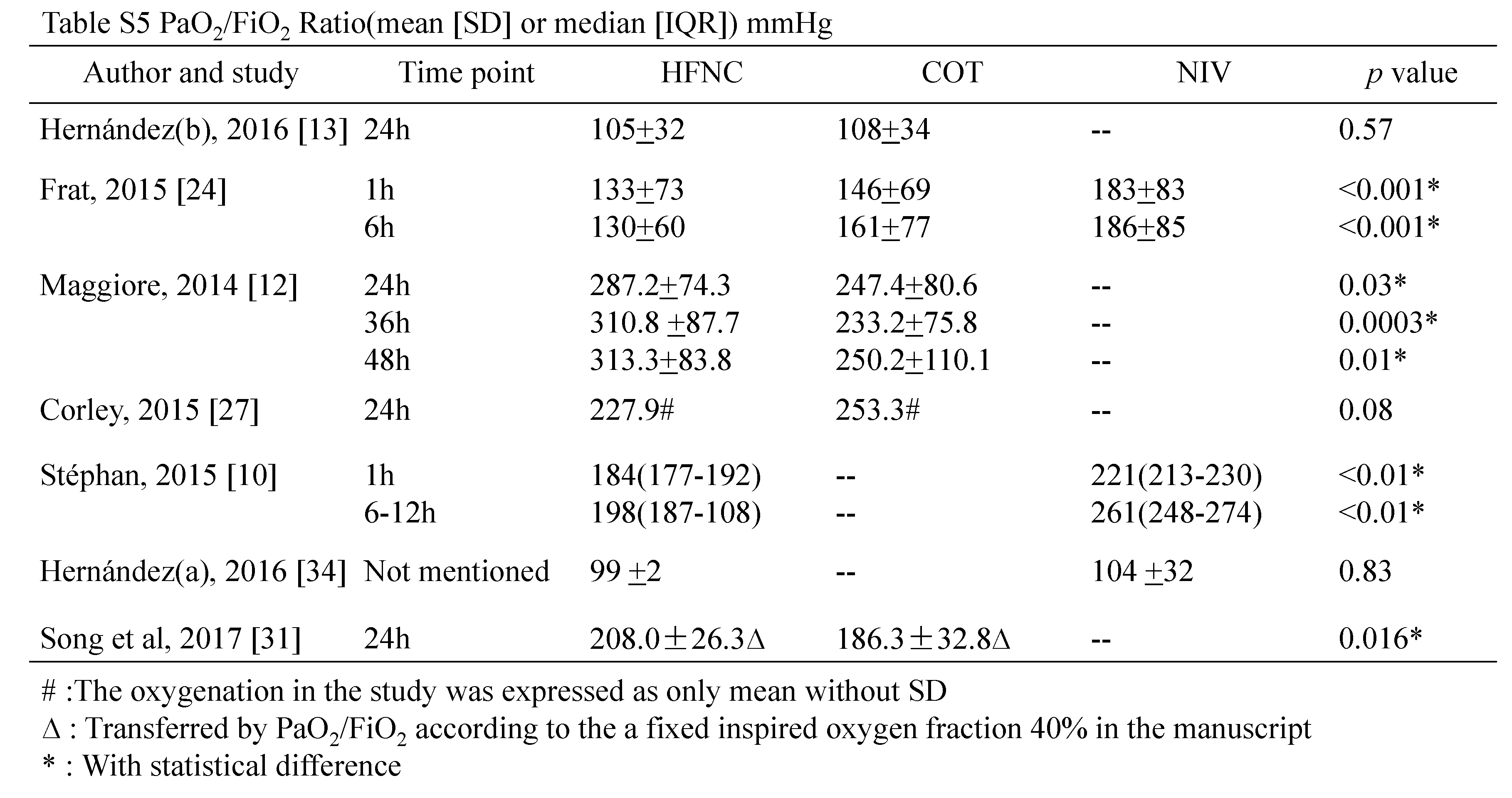

Supplement: Supplementary file 2 — Additional Tables and Figures. (ZIP 5033 kb) [file 12931_2018_908_MOESM2_ESM.zip › table S5 Oxygenation.tif]
